# Recurrent swelling of right knee: a case of hemangioma

**DOI:** 10.1186/1546-0096-9-S1-P45

**Published:** 2011-09-14

**Authors:** J Wipff, H Guerini, C Deslandre

**Affiliations:** 1 Rheumatology A Department; 2 Radiology B, Cochin Hospital, Paris Descartes University, APHP, Paris, France

## Case report

We report the case of a 2-year girl who presented, in 2007, lameness with swelling of her right knee. Her leg has been putted in plaster but pain remained intense one month after. Blood tests did not show any inflammatory syndrome and standards radiographies were normal. Synovial fluid ponction and synovial biopsy were performed and confirmed a non specific synovitis. These results led to evocate a juvenile oligoarthritis despite the absence of antinuclear antibodies and uveitis. Treatment with Non Steroid Anti-Inflammatory Drugs (NSAIDs) allowed remission of symptoms.

Symptoms reappeared 2 years later with swelling of the right knee associated with a localized tumefaction of the external part of the patella. Standard radiographies showed a hypertrophic patella due to abnormal growth. New complete investigations invalidated the diagnosis of oligoarthritis. Right knee echography showed synovitis in the sub-quadricipital recessus and a mass with several tortuous lesions which evocated 	a hemangioma in Hoffa’s fat. Another ultrasound confirmed that this hemangioma was at the expense of the lateral chief of quadriceps.

Currently, this young girl is treated by NSAIDs and an interventional treatment by sclerosis first and then by surgery is discussed but the age of this patient leads to delay these treatments.

## Discussion

Vascular malformations are divided into various subcategories depending on the predominant anomalous channels: venous, lymphatic, capillary and arterial. Prevalence of hemangiomas is estimated at 1.5% in general population.

Clinical symptoms (pain, oedema, hemorrhagic) depend on their localisation (muscular, osseous) and their local extension. The main localisations of hemangiomas are: pelvis, limbs and cranial circulation.

Venous malformation are the most frequent (2/3 cases). They can be localized, as in our patient, multifocal or diffuse.

The best imageries to diagnose and describe hemangiomas are MRI and ultrasound with colour Doppler. In MRI, the aspect of the lesion is in isosignal T1 and hypersignal T2. Ultrasound allows the localisation of the mass and the visualisation of tortuous lesions evocating hemangioma.

Medical treatment is often insufficient and needs to be completed with interventional procedures: sclerotherapy for diffuse lesion or surgery for localized mass. Combination of these two techniques is, in some cases, necessary.

Prognosis is largely favorable without sequellae.

Recurrent swelling of the knee is not necessarily due to idiopathic juvenile arthritis but can be due to other local diagnosis as hemangioma. In atypical symptoms or evolution, ultrasound and/or MRI is helpful to rectify the diagnosis.

